# A circular dichroism study of the protective role of polyphosphoesters polymer chains in polyphosphoester‐myoglobin conjugates

**DOI:** 10.1002/chir.23486

**Published:** 2022-06-17

**Authors:** Chiara Pelosi, Lorenzo Arrico, Francesco Zinna, Frederik R. Wurm, Lorenzo Di Bari, Maria R. Tinè

**Affiliations:** ^1^ Dipartimento di Chimica e Chimica Industriale Università di Pisa Pisa Italy; ^2^ Sustainable Polymer Chemistry (SPC), Department of Molecules and Materials, MESA+ Institute for Nanotechnology, Faculty of Science and Technology University of Twente Enschede Netherlands

**Keywords:** circular dichroism, polyphosphoesters, protein thermal stability, protein unfolding, protein‐polymer conjugates

## Abstract

Protein‐polymer conjugates are a blooming class of hybrid systems with high biomedical potential. Despite a plethora of papers on their biomedical properties, the physical–chemical characterization of many protein‐polymer conjugates is missing. Here, we evaluated the thermal stability of a set of fully‐degradable polyphosphoester‐protein conjugates by variable temperature circular dichroism, a common but powerful technique. We extensively describe their thermodynamic stability in different environments (in physiological buffer or in presence of chemical denaturants, e.g., acid or urea), highlighting the protective role of the polymer in preserving the protein from denaturation. For the first time, we propose a simple but effective protocol to achieve useful information on these systems in vitro, useful to screen new samples in their early stages.

## Graphical abstract

- Variable temperature circular dichroism as accessible technique to sceen drug stability
- Investigation of thermal stability of fully‐degradable protein‐polymer conjugates
- The protective role of biodegradable polyphosphoesters toward protein in protein‐polymer conjugates

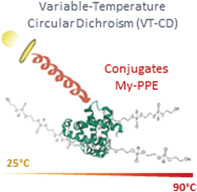

## 1 INTRODUCTION

Protein‐polymer conjugates are hybrid systems where a protein is covalently bound to synthetic polymer chains. Such systems express high potential in the biomedical field, thanks to their ability to enhance the lifetime of protein‐based drugs in the human body, as a result of the increased drug radius, the decreased protein aggregation, and the reduction of the drug recognition and clearance by the immune system.^1^, ^2^ Besides, the bioconjugation strategy often leads to an enhancement of the protein stability in solution.^3^, ^4^

The most common polymer used to synthesize protein‐polymer conjugates is poly (ethylene glycol) (PEG), which is currently used in 15 protein‐polymer conjugates‐based formulations available on the market.^2^ In the last 20 years, in spite of their great use, PEGylated drugs have shown some drawbacks linked to the accumulation of PEG in the body of patients after long‐term treatments, and to the discovery of anti‐PEG antibodies.^5^, ^6^, ^7^ These issues pushed the search for PEG alternatives, for example, polyoxazolines, polyglycerol, and polypeptides.^2^, ^8^, ^9^ Further, water‐soluble polyphosphoesters (PPEs) are a class of biodegradable and biocompatible polymers recently proposed as a valuable PEG alternative in biomedicine,^8^, ^10^ used to develop drug nanocarriers,^11^ surfactants,^12^ hydrogels,^8^ anti‐fouling coatings,^13^ protein‐polymer conjugates,^14^, ^15^, ^16^ and so on.

Despite the importance of the topic, an in‐depth study on the influence of the polymer on the conjugates' stability is often missing in the study of new structures. Generally speaking, the most powerful techniques used to assess the thermodynamic parameters related to the protein unfolding process are nano‐differential scanning calorimetry (n‐DSC),^17^, ^18^ nano‐differential scanning fluorimetry (n‐DSF),^19^ and circular dichroism (CD).^20^ The latter allows a rapid determination of the protein secondary structure (e.g., α‐helices, β‐sheets, or random coils), as different structural elements exhibit characteristic CD spectra.^21^, ^22^ Hence, with CD spectroscopy the variations of the protein secondary structure and the thermodynamic parameters associated to the protein unfolding induced by heat, osmolytes, denaturants, or ligands can be studied.^20^, ^21^, ^23^ Also, in contrast to the other above‐mentioned techniques, CD studies can be performed on very diluted protein samples (up to 10^−6^ M, vs. 10^−5^/10^−4^ for n‐DSF and n‐DSC). This minimizes the post‐unfolding aggregation phenomena typical of many globular proteins, which often hamper the correct thermodynamic evaluation of the protein unfolding, possible only in reversible conditions.^24^

In this work, we used variable temperature CD (VT‐CD) to investigate the thermal stability in solution of a set of PPE‐modified, (PPEylated) proteins constituted of myoglobin and a series of PPEs varying in hydrophilicity, compared to a PEGylated myoglobin as a reference. The analysis allowed us to use thermodynamic models for describing protein unfolding, and to calculate the enthalpy, the melting temperature, and the Gibbs energy of the denaturation process. The denaturing action of sulfuric acid and urea on the protein thermal stability was also evaluated. This investigation permitted us to test the sample response in different environments, which can simulate real conditions (e.g., protein denaturation induced by acid pH around tumoral cells). Besides, the comparison between the denaturing action of urea and sulfuric acid, which induce denaturation through different mechanisms, gave insight into the polymer's role as a modulator of the protein thermodynamic stability.

The protein chosen to form the conjugates was myoglobin from equine skeletal muscle, since it has a well‐known globular structure with lysine residues on the surface available for the bioconjugation reaction. In addition, the heme group of myoglobin is responsible for a Soret band in the UV–Vis spectra in the range 350–450 nm, which can be used as a reporter of protein conformational changes around the active site, as shown in previous publications.^25^, ^26^ Also, the myoglobin test structure, dynamics and thermal stability have been widely studied by means of different spectroscopies.^25^, ^27^, ^28^

## 2 MATERIALS AND METHODS

### 2.1 Materials

Dulbecco's phosphate‐buffered saline without calcium and magnesium was purchased from Thermo Fisher Scientific and used as received. Sulfuric acid (95%–97% purity) and urea (ACS reagent, 99%) were purchased from Sigma Aldrich (Italy). The protein‐polymer conjugates studied in this work were synthesized and characterized in one of our previous works.^16^ More in detail, different protein‐polymers conjugates were obtained, by covalently attaching myoglobin from equine skeletal muscle (My, Sigma Aldrich, 99.5% purity) to polyethylene glycol (PEG, 10 kDa, Thermo Fisher Scientific), and to poly (methyl ethylene phosphonate) (PMeEP) or poly (ethyl‐co‐butyl ethylene phosphonate) (PEtEP‐*co*‐BuEP), with a molecular weight of 10 kDa, synthesized by anionic‐ring opening polymerization, as described in our previous work.^16^ In all the conjugates ~3 polymer chains of 10 kDa each are attached to the protein. The following notation is used in the text: My is the neat protein, My‐PEG, My‐PMeEP, My‐PEtEP‐*co*‐BuEP are the conjugates in which each protein is attached to ~3 chains of respectively PEG, PMeEP, PEtEP‐*co*‐BuEP with a molecular weight of 10 kDa.

### 2.2 Circular dichroism measurements

CD measurements were performed using a Peltier apparatus (Jasco PTC‐517) in the range 190–500 nm with a Jasco J1500 spectropolarimeter. The spectra were collected by performing four scans at 100 nm/min, using a concentration of ~1.5 × 10^−5^ M (0.25 mg/ml) of protein within the sample, in DPBS, in a 1‐mm cell at room temperature. The spectra were background‐corrected using the DPBS spectrum recorded under the same conditions. The effective protein concentration was estimated for each sample by measuring the absorbance at 290 nm in a 1‐cm cell, considering an *ε* of 1.422 (mg ml)^−1^ at 290 nm. The protein secondary structure in the conjugates was evaluated by comparing the spectra of the samples between 200 and 500 nm, due to the buffer cutoff (lower wavelength limit for DPBS = 200 nm).^20^ VT‐CD experiments were performed in a 1‐cm cell by monitoring the CD signal at 222 nm while heating the samples at a constant scan rate of 1°C/min, from 25°C to 90°C, using an internal temperature probe. The spectra were background‐corrected using DPBS signal recorded under the same conditions. The measurements for the samples alone were recorded using a concentration of ~1.5 × 10^−6^ M (0.025 mg/ml) of protein within the sample, in DPBS. The measurement in presence of denaturant (acid or urea) were recorded using a sample concentration of ~1 x 10^−6^ M, adding sulfuric acid (0.01 M) or urea (with a final concentration of respectively 1 * 10^−3^ M and 0.5 M and a molar ratio denaturant/protein of respectively 2 * 10^3^ and 5 * 10^5^), until the obtaining half of the initial CD signal intensity. The spectra of the samples denatured with urea were recorded after at least 2 h of urea addition, to ensure the reaction completion. The thermodynamic analysis of the data was performed using OriginPro8 SR4 (version 8, Origin Lab Corporation). In brief, we fitted the experimental data with a two‐state unfolding model, assuming that the heat capacity drop was not dependent from the temperature. More detail on the fitting equation used are reported in the supporting information, paragraph 1.

## 3 RESULTS AND DISCUSSION

**FIGURE 1:**
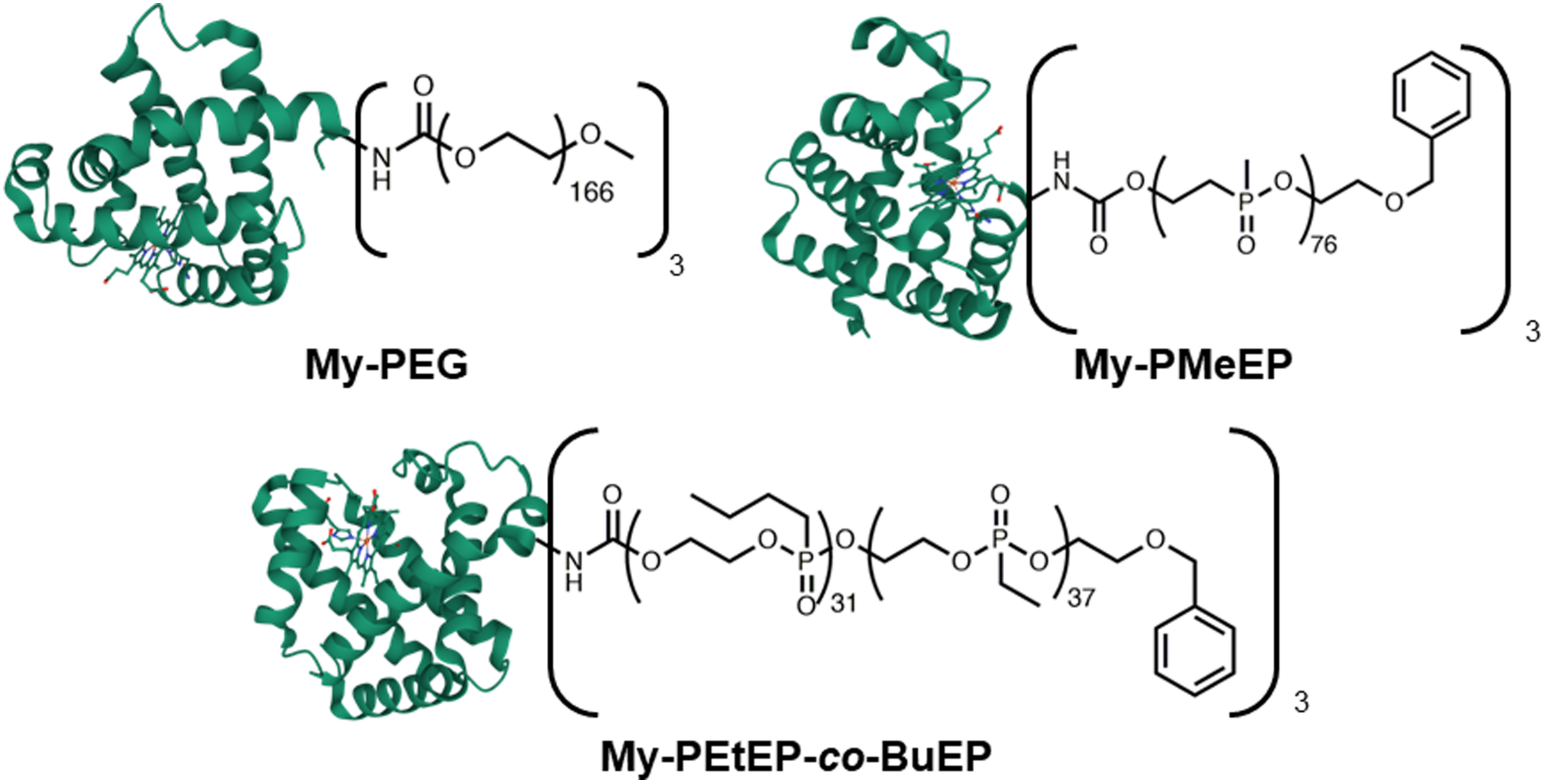
Illustration of the conjugates under study. Image of myoglobin was taken from the RCSB PBD (rcsb.org) of PDB ID 5CN5 (Barends, T.R., et al.) (2015) Direct observation of ultrafast collective motions in CO myoglobin upon ligand dissociation. Science 350: 445–450^29^

An extensive evaluation of the protein unfolding of a series of PPEylated protein‐polymer conjugates was conducted via CD spectroscopy in an aqueous solution. The conjugates under study (Figure 1) are composed of myoglobin with approximately three chains of PMeEP or PEtEP‐*co*‐BuEP (two water‐soluble PPEs with different hydrophilicity) covalently attached. We also evaluated, for comparison, the PEGylated conjugate (My‐PEG) synthesized with an analogue procedure.

**FIGURE 2:**
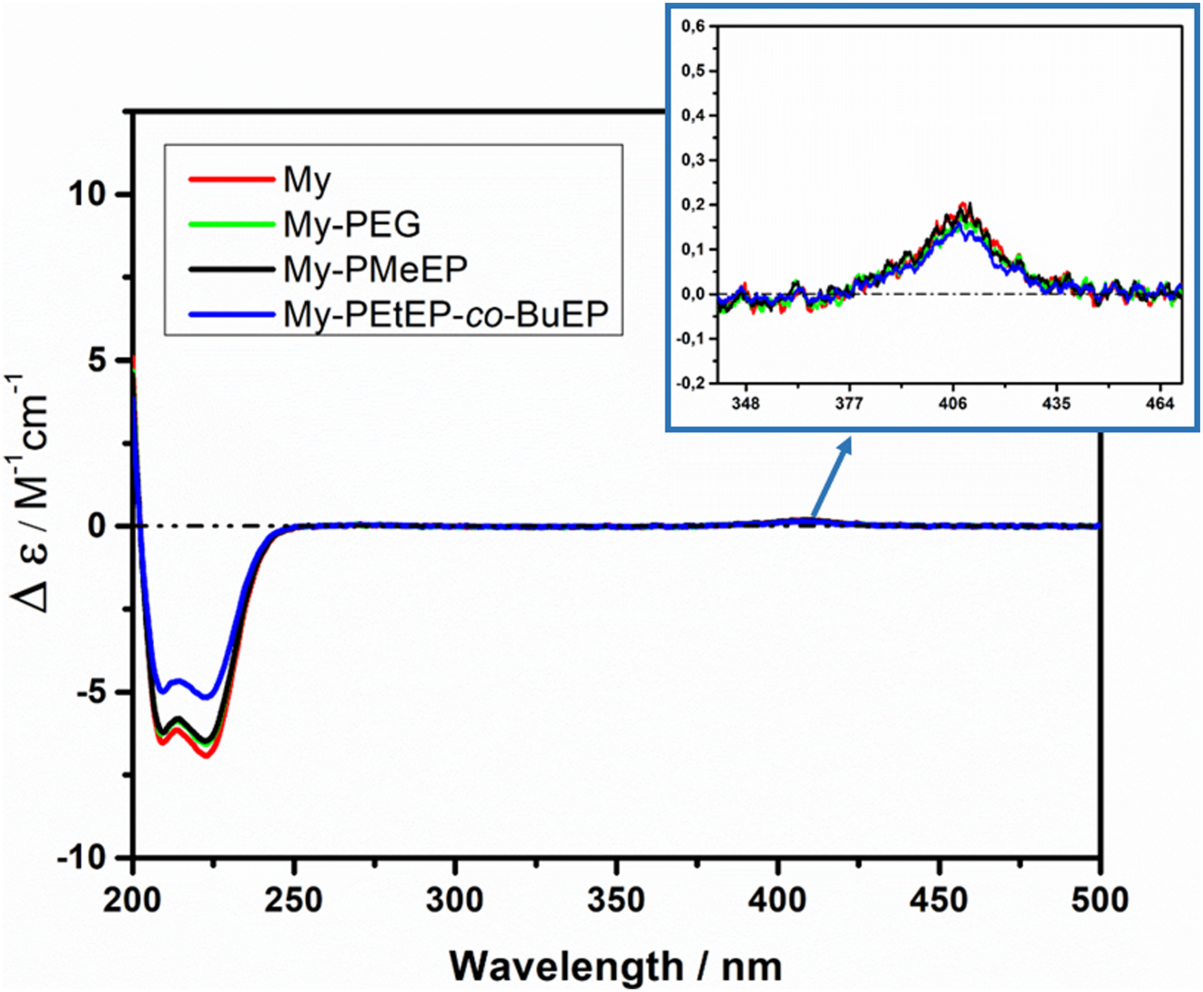
Circular dichroism (CD) spectra of the conjugates and native My with an expansion in the region between 340 and 470 nm

Firstly, we evaluated the secondary structure of myoglobin in an aqueous solution at 25°C. Due to the cutoff of the medium used to prepare proteins solutions, we obtained significant CD data starting from 200 nm.^20^ The CD spectra showed a strong negative signal with two minima at 209 and 222 nm (Figure 2), which stems from the α‐helix domains of the protein, in accordance with the data reported in the literature.^27^, ^30^ Also, an additional small positive signal with the maximum at 409 nm was present, associated to the protein heme group. The spectrum of native protein exhibited a profile superimposable to the conjugates My‐PEG and My‐PMeEP in the α‐helix region, suggesting that the bioconjugation process and the polymer chains presence did not significantly affect the secondary structure of the proteins. On the other hand, the conjugate My‐PEtEP‐*co*‐BuEP presented a slight decrease of α‐helix content, as indicated by the decrease of the CD signal intensity. Previously, we reported that the bioconjugation procedure used in this work (the synthesis and purification of the conjugates) did not induce any alteration of the protein secondary and tertiary structure^16^; thus, we believe that the changes observed were due to a slight modification of the protein structure induced by the establishment of interactions among the protein and the polymer chains. The conjugate with PEtEP‐*co*‐BuEP (the more hydrophobic polymer) showed a higher destabilization compared to more hydrophilic PEG and PMeEP.^26^ This result was in line with evidence reported in one of our previous work,^26^ which highlighted the dependence of some of the conjugates properties on the hydrophilicity of the attached polymer. In particular, it was observed that^26^ (i) the protein residue activity decreased with decreasing the hydrophilicity of the attached polymer (Residue activity; My‐PEG: 86%, My‐PMeEP: 85%, My‐PEtEP‐*co*‐BuEP: 73%, compared to the activity of native myoglobin set to 100%); (ii) the surrounding of the protein heme group was slightly altered by the presence of the more hydrophobic polymers.

**FIGURE 3:**
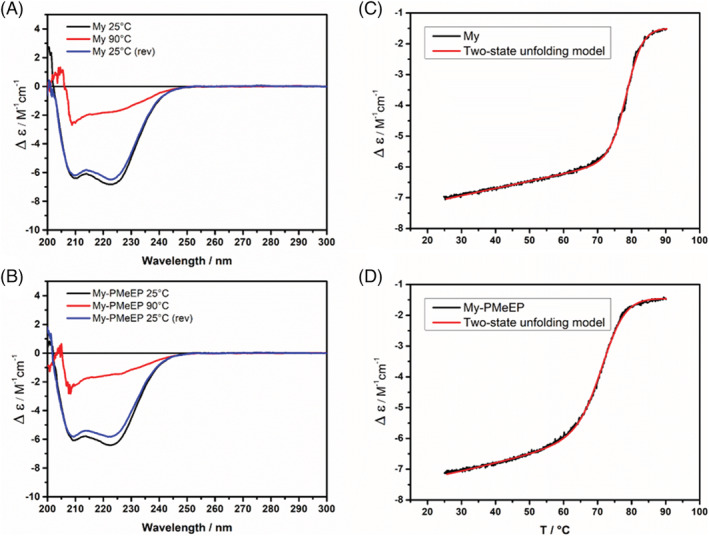
CD spectra of (A) My and (B) My‐PMeEP at 25°C (black curve), 90°C (red curve), and at 25°C after cooling (blue curve). Thermal denaturation profiles of (C) My and (D) My‐PMeEP performed by variable temperature circular dichroism (VT‐CD) measurements, monitoring the CD signal at 222 nm during a heating scan at 1°C/min from 25°C to 90°C. The red line is the theoretical curve calculated using a two‐state unfolding model. The spectra of the other samples are reported in Figure S1

Concerning the samples' thermal stability, we monitored the variation of the CD signal at a fixed wavelength (222 nm) during a heating scan at a constant scan rate (1°C/min) (Figures 3 and S1). We also registered the CD spectra in the range 200–300 nm at 25°C, 90°C, and again at 25°C after the sample cooling. The percentage ratio among the CD values recorded at 222 nm after and before the thermal scan was defined as the sample's reversibility index (RI %), which accounts of the percentage of protein correctly and completely refolded in the experimental conditions.^31^ The protein myoglobin exhibited a RI% of 90% (Table 1), higher than the value that we reported in one of our previous papers, recorded by n‐DSC in the same buffer at concentrations ca 100 times higher (RI%: 26%).^26^ We previously explained the low reversibility obtained by n‐DSC with the occurrence of post unfolding aggregation phenomena, typical of many globular proteins. The increasing of reversibility obtained for native myoglobin in the present work endorses the hypothesis previously made, because the VT‐CD measurements were conducted at a low protein concentration. The same trend was observed for the unfolding reversibility of My‐PEt‐*co*‐BuEP (RI% previously obtained by n‐DSC: 37%^26^; RI% obtained by VT‐CD: 87%). In the same work, we also observed that the more hydrophilic polymers PEG and PMeEP increased the protein reversibility even at the concentrations used by n‐DSC (RI% of the respective conjugates obtained by n‐DSC: 81%; 83%)^26^; in this case, we believed that the polymers could form a shield around the protein, hampering the inter‐molecular interactions among hydrophobic residues of the protein in the unfolded form, which cause the formation of aggregates and precipitates. Comparing these values with the results obtained by VT‐CD (RI% of 92% and 88% respectively), we believe that in the latter, the high values obtained could be given by a combination of two different effects: the dilution effect and the protective action of the hydrophilic polymers.

**TABLE 1:** Thermodynamic parameters obtained from the analysis of thermal denaturation of My and the conjugates My‐PEG, My‐PMeEP, and My‐PEtEP‐co‐BuEP alone or in presence of sulfuric acid or urea

Thermal unfolding^a^					
	*T*_onset_ (°C)^b^	Δ_d_ *H*° (kJ/mol)^c^	*T*_m_ (°C)^c^	Δ_d_ *G°* (25°C) (kJ/mol)^d^	RI %^e^
My	72	454	79	30	90%
My‐PEG	68	349	74	16	92%
My‐PMeEP	63	276	70	7	88%
My‐PEtEP‐*co*‐BuEP	46	175	58	0.4	87%

Thermal unfolding with H_2_SO_4_ ^a^					
My	41	193	65	−1	84%
My‐PEG	67	323	75	11	83%
My‐PMeEP	64	262	73	4	81%
My‐PEtEP‐*co*‐BuEP	42	144^f^	57^f^	−2^f^	18%

Thermal unfolding with urea^a^					
My	70	345	77	14	89%
My‐PEG	65	299	71	9	82%
My‐PMeEP	59	245	68	4	72%
My‐PEtEP‐*co*‐BuEP	42	163	52	1	78%

*Note*: The experimental curves were fitted with a two‐state unfolding model.

^a^ Data reported as means values of at least three measurements, with the following error bars: *T*_onset_: ±1°C for thermal unfolding, ±2°C for thermal unfolding with H_2_SO_4_ or urea; Δ_d_ *H*°: <35KJ/mol; *T*_m_: ±1°C; Δ_d_G°: RDS <15%; RI%: <7%.

^b^ Calculated from the experimental curve, as intersection between the extrapolated baseline and the tangent passing by the first inflexion point.

^c^ Best‐fit parameters calculated applying a two‐state unfolding model.

^d^ Gibbs energy calculated at 25°C using Equation (S2).

^e^ Calculated as described in Equation (S1).

^f^ The measures were largely affected by concomitant aggregation effect; thus, the results are indicative and reported only for sake of comparison.

The overall comparison among the data obtained on the conjugates by n‐DSC^26^ and VT‐CD highlights the great advantage in the use of the latter to study samples with concentration dependent behavior. In fact, it permits to perform the measurements at protein concentrations down to 10^−6^ M (a hundred times lower than the values commonly used with other techniques^20^).

The second step was the thermodynamic analysis of the process. Based on previous results obtained for similar samples,^32^ we assumed that the small part of irreversible processes (≈10%) occurred close to the end of the heating scan (around 90°C). This allowed the use of thermodynamic models to describe the unfolding process, as we considered the overall thermal profile not significantly affected by these phenomena.^33^ Therefore, we applied a deconvolution procedure, fitting the experimental curves with a two‐state unfolding model, following a literature procedure to determine by VT‐CD the thermodynamics parameters of the protein unfolding.^20^ More details on the fitting equations used are reported in Equations S1–S5.

**FIGURE 4:**
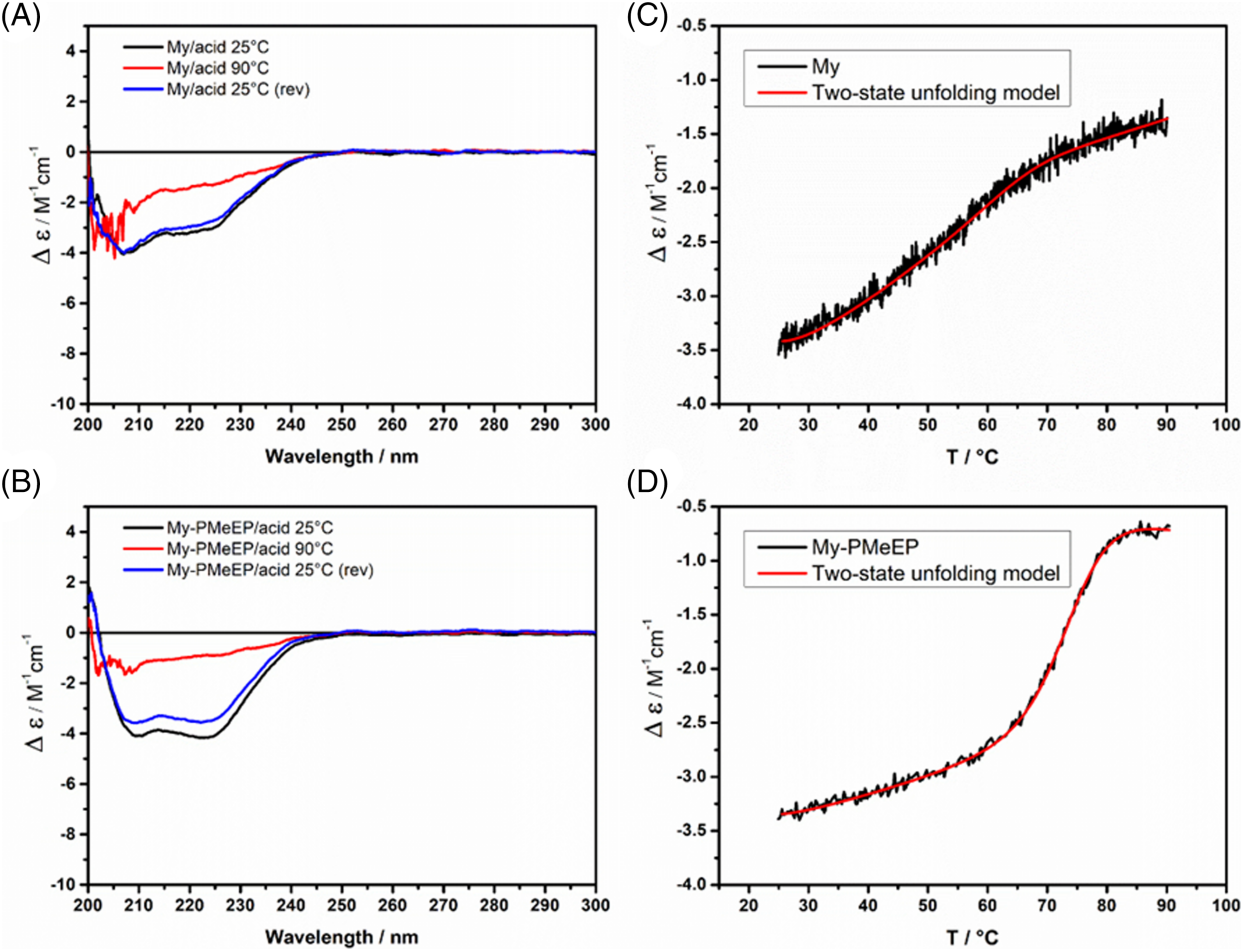
Circular dichroism (CD) spectra of (A) My and (B) My‐PMeEP at 25°C (black curve), 90°C (red curve), and at 25°C after cooling (blue curve) in presence of sulfuric acid (final sulfuric acid concentration: 1 * 10^−3^ M; molar ratio denaturant/protein 2 * 10^3^). Thermal denaturation profiles of (C) My and (D) My‐PMeEP in presence of sulfuric acid, performed by variable temperature CD (VT‐CD) measurements, monitoring the CD signal at 222 nm during a heating scan at 1°C/min. The red line is the theoretical curve calculated using a two‐state unfolding model. The spectra of the other samples in presence of acid and urea are reported in Figures S2 and S3

We further investigated the thermal unfolding of the samples when this process is affected by the presence of chemical denaturants (sulfuric acid and urea). The VT‐CD heating scans recorded at a fixed wavelength (222 nm) are reported in Figures 4, S2, and S3. The reversibility of the protein unfolding showed a slight decrease in all the cases, except for My‐PEtEP‐*co*‐BuEP with sulfuric acid, which showed a high drop in reversibility (Table 1). Beyond the latter, we considered the thermograms not significantly affected by the low percentages of irreversible processes, in analogy with the study of the process in absence of denaturants reported above. The curves were satisfactorily fitted with a two‐state model, revealing that the protein unfolding mechanism is independent from the use of denaturants. In the case of My‐PEtEP‐*co*‐BuEP in presence of sulfuric acid, being the RI % of only 18%, the data could not be treated with thermodynamic models, hence the fitting was performed only as a comparison with the other samples.

Table 1 gathers the thermodynamic data obtained with the fitting procedure performed on the samples in solution alone or in presence of the denaturant (sulfuric acid or urea). In particular, we reported the *T*_onset_ and *T*_m_ (respectively the temperatures at which the unfolding begins and at which 50% of the protein is unfolded), the denaturation enthalpy (Δ_d_ *H*°), the Gibbs energy (Δ_d_ *G°)* calculated at 25°C, and the RI%.

In the case of the thermal unfolding in absence of denaturants, we observed that the presence of the polymer led to a thermodynamic protein destabilization (lower Δ_d_ *H*°, *T*_onset_, and *T*_m_). We observed the same trend also for the values of standard Gibbs free energy calculated at 25°C, which were not further discussed due to the error affecting the experimental data. This destabilization was linked to the polymer hydrophilicity: the more hydrophobic polymer PEtEP‐*co*‐BuEP induced a more pronounced protein destabilization, in accordance with the literature.^26^ In addition, this result was in line with the changes in the protein secondary structure observed above, which suggest its partial reorganization within the conjugate at room temperature, explaining the low energy amount required to complete the unfolding process. It is worth mentioning that the profile of My‐PEtEP‐*co*‐BuEP was successfully described with a two‐state unfolding model. We believe that the main reason for our success was the low concentration of protein in the measurement conditions, allowed by CD, which minimized the post‐unfolding aggregation phenomena. On the contrary, studies performed with other analytical techniques, such as n‐DSF and n‐DSC did not allow the application of any theoretical model to this system, as it was plagued by aggregation phenomena concomitant to the unfolding process, significant in the conditions where these measurements were performed.^26^

The effect of the denaturants on the protein unfolding was investigated more in detail by calculating the differences of the parameters obtained in absence and in presence of the denaturants. The results are reported in Table 2.

**TABLE 2:** Variation of *T*_onset_, Δ_d_ *H*°, and *T*_m_ of the protein unfolding process in presence of the denaturants urea or sulfuric acid

Sample	With urea^a^			With acid^a^		
	Δ*T*_onset_ (°C)^b^	ΔΔ_d_ *H*° (kJ/Mol)^c^	Δ*T*_m_ (°C) ^b^	Δ*T*_onset_ (°C) ^b^	ΔΔ_d_ *H*° (kJ/Mol) ^c^	Δ*T*_m_ (°C) ^b^
My	−2	−109	−3	−31	−261	−14
My‐PEG	−3	−50	−3	−1	−26	0
My‐PMeEP	−4	−31	−3	−0	−14	0
My‐PEtEP‐*co*‐BuEP	−4	−12	−6	−4	−31	−1

^a^ The values are reported as difference between the parameter obtained in presence of denaturant and the one obtained in absence of denaturant.

^b^ Error ±3°C.

^c^ Error <70 kJ/mol.

Table 2 clearly shows that the presence of sulfuric acid influences heavily the myoglobin unfolding parameters, by reducing *T*_onset_, Δ_d_ *H*°, and *T*_m_ of respectively 31°C, 261 kJ/mol, and 14°C. The effect was strongly reduced in the conjugates, which presented negligible variations. We hypothesized that the polymers acted as a proton scavenger, possibly thanks to interactions between the oxygens in the chains and the hydrogen ions, which would protect the protein from acid denaturation. The protection against the acidic environment is a noticeable result because it simulates particular body conditions, for example, the protein denaturation which may occur in physio‐pathological environment in the vicinity to tumoral cells. Protein denaturation induced by urea was also slightly reduced by the polymer presence. In this case, we suppose that the polymer reduced the interaction with the protein by steric hindrance. It is worth noticing that the polymer hydrophilicity had an influence also on the protective action toward the protein, with the more hydrophilic polymers showing the best performances. In fact, the polymer PMeEP (more hydrophilic than the copolymer PEtEP‐*co*‐BuEP) showed higher protein preservation, exploiting a function comparable to the PEGylated analogue.

## 4 CONCLUSION

In this work, we propose the use of variable‐temperature CD as a highly accessible technique to derive information on the thermal stability of protein‐polymer conjugates in different environments. As proof of concepts, we reported an extensive evaluation of a set of conjugates constituted by the protein myoglobin and polymers belonging to the promising class of biodegradable PPEs. The protein unfolding process was successfully described with a two‐state unfolding model both for the native and the PPE‐conjugates protein. While conjugation would not seem to significantly alter the conformation and the stability of the protein in physiological conditions, we discovered a noticeable protective action of the polymer toward two common denaturants: acid environment and urea. The reliability of the data, together with the advantages offered by CD (easy to use, quick measurements, possibility to work at low concentrations and to obtain information on the protein secondary structure) render the proposed methodology appealing to screen in vitro new conjugates with therapeutical applications, orienting future studies in the clinics.

## Supporting information


**Figure S1:** CD spectra of a) My‐PEG and b) My‐PEtEP‐co‐BuEP at 25 °C (black curve), 90 °C (red curve), and at 25 °C after cooling (blue curve). Thermal denaturation profilesvof (c) My‐PEG and d) My‐PEtEP‐co‐BuEP performed by VT‐CD measurements, monitoring the CD signal at 222 nm during a heating scan at 1 °C/min. The red line is the theoretical curve calculated using a two‐state unfolding model.
**Figure S2:** CD spectra of a) My‐PEG and b) My‐PEtEP‐co‐BuEP at 25 °C (black curve), 90 °C (red curve), and at 25 °C after cooling (blue curve) in presence of sulphuric acid. Thermal denaturation profiles (black line) of (c) My‐PEG and (d) My‐PEtEP‐co‐BuEP in presence of sulphuric acid (final sulfuric acid concentration: 1*10^−3^ M; molar ratio denaturant/protein 2*10^3^), performed by VT‐CD measurements, monitoring the CD signal at 222 nm during a heating scan at 1 °C/min. The red line is the theoretical curve calculated using a two‐state unfolding model. In the case of My‐PEtEP‐co‐BuEP in presence of sulfuric acid, being the RI % of only 18%, the data could not be treated with thermodynamic models, hence the fitting was performed only as a comparison with the other samples.
**Figure S3:** CD spectra of a) My, b) My‐PMeEP, c) My‐PEG and d) My‐PEtEP‐co‐BuEP at 25 °C (black curve), 90 °C (red curve), and at 25 °C after cooling (blue curve), in presence of urea. Thermal denaturation profiles of e) My, f) My‐PMeEP, g) My‐PEG and h) My‐PEtEP‐co‐BuEP in presence of urea (final urea concentration: 0.5 M; molar ratio denaturant/protein 5*10^5^), performed by VT‐CD measurements, monitoring the CD signal at 222 nm during a heating scan at 1 °C/min. The red line is the theoretical curve calculated using a two‐state unfolding model.

## Data Availability

Data available on request from the authors.
