# Supplementary material for: A circular dichroism study of the protective role of polyphosphoesters polymer chains in polyphosphoester‐myoglobin conjugates
Source: Chirality. 2022 Jun 17;34(9):1257–65. doi: 10.1002/chir.23486 (PMC9544571; doi:10.1002/chir.23486)
Supplement: Supplementary file 1 — Figure S1: CD spectra of a) My‐PEG and b) My‐PEtEP‐co‐BuEP at 25 °C (black curve), 90 °C (red curve), and at 25 °C after cooling (blue curve). Thermal denaturation profilesvof (c) My‐PEG and d) My‐PEtEP‐co‐BuEP performed by VT‐CD measurements, monitoring the CD signal at 222 nm during a heating scan at 1 °C/min. The red line is the theoretical curve calculated using a two‐state unfolding model. Figure S2: CD spectra of a) My‐PEG and b) My‐PEtEP‐co‐BuEP at 25 °C (black curve), 90 °C (red curve), and at 25 °C after cooling (blue curve) in presence of sulphuric acid. Thermal denaturation profiles (black line) of (c) My‐PEG and (d) My‐PEtEP‐co‐BuEP in presence of sulphuric acid (final sulfuric acid concentration: 1*10−3 M; molar ratio denaturant/protein 2*103), performed by VT‐CD measurements, monitoring the CD signal at 222 nm during a heating scan at 1 °C/min. The red line is the theoretical curve calculated using a two‐state unfolding model. In the case of My‐PEtEP‐co‐BuEP in presence of sulfuric acid, being the RI % of only 18%, the data could not be treated with thermodynamic models, hence the fitting was performed only as a comparison with the other samples. Figure S3: CD spectra of a) My, b) My‐PMeEP, c) My‐PEG and d) My‐PEtEP‐co‐BuEP at 25 °C (black curve), 90 °C (red curve), and at 25 °C after cooling (blue curve), in presence of urea. Thermal denaturation profiles of e) My, f) My‐PMeEP, g) My‐PEG and h) My‐PEtEP‐co‐BuEP in presence of urea (final urea concentration: 0.5 M; molar ratio denaturant/protein 5*105), performed by VT‐CD measurements, monitoring the CD signal at 222 nm during a heating scan at 1 °C/min. The red line is the theoretical curve calculated using a two‐state unfolding model. [file CHIR-34-1257-s001.docx]

***SUPPORTING INFORMATION***

A circular dichroism study of the protective role of polyphosphoesters (PPE) polymer chains in PPE-myoglobin conjugates.

Chiara Pelosi,*^[a]^ Lorenzo Arrico,^[a]^ Francesco Zinna,* ^[a]^ Frederik R. Wurm,^[b]^ Lorenzo Di Bari, ^[a]^ and Maria R. Tinè.^[a]^

[a] Dipartimento di Chimica e Chimica Industriale, Università di Pisa, Via Moruzzi 13, 56120, Pisa (Italy).

[b] Sustainable Polymer Chemistry (SPC), Department of Molecules and Materials, MESA+ Institute for Nanotechnology, Faculty of Science and Technology, University of Twente, P.O. Box 217, 7500 AE Enschede (Netherlands).

*Corresponding authors e-mails: [chiara.pelosi@dcci.unipi.it](mailto:chiara.pelosi@dcci.unipi.it); [francesco.zinna@unipi.it](mailto:francesco.zinna@unipi.it)

**TABLE OF CONTENT:**

1. **CD DATA TREATMENT**
2. **CD SPECTRA**
3. **REFERENCES**
4. **CD DATA TREATMENT**

Variable temperature circular dichroism (VT-CD) was used to describe the thermodynamic of the protein unfolding within the conjugates. An extensive treatment of the theoretical background was previously described in other papers.^1–3^ Here, we report the equations used to perform the biophysical analysis of the experimental data.

The CD spectra in the range 200-300 nm were recorded at room temperature (25°C). Then, VT-CD signal were recorded at a fixed wavelength (222 nm) during a heating scan from 25°C to 90°C with a constant heating rate (1°C/min). The collected data were fitted with a two-state unfolding model, using a fixed *ΔC*_p_ taken from the literature (*ΔC*_p_ = 7680 J/mol K),^4^ thus assuming that the heat capacity change was not dependent on temperature. In particular, we used the following equations:^3^

$\Delta G=\Delta H*\left( 1-\frac{T}{T_{m}} \right)-(\Delta C_{p}*\left( T-T_{m} \right)+(T* \ln\frac{T}{T_{m}}))$ (Eq. S1)

$K=e^{\frac{-\Delta G}{8.32*T}}$ (Eq. S2)

$\theta_{n}=(p*T)+q$ (Eq. S3)

$\theta_{d}=(m*T)+n$ (Eq. S4)

${\Delta C}_{p}= \frac{K}{1-K}*\left( \theta_{d}- \theta_{n} \right)+ \theta_{n}$ (Eq. S5)

Where T (expressed in kelvin) is the observed temperature, T_m_ (expressed in kelvin) is the temperature at which 50% of the protein is unfolded, ΔH (expressed in kJ/mol) is the unfolding enthalpy, ΔG (expressed in kJ/mol) is the unfolding free energy, K is the equilibrium constant, θ_n_ and θ_d_ are respectively the ellipticity of the native and unfolded protein, which depend linearly from temperature as described in the equations S2 and S3. Besides, the onset temperature (*T*_onset_) was calculated at the intersection between the extrapolated baseline and the tangent passing by the first inflection point.

After the heating scan, the CD spectra in the range 200-300 nm at 90°C were recorded, then the sample was allowed to return to room temperature (25°C) and a new spectrum in the same wavelength range was recorded. The reversibility index percentage (RI%) was calculated as described in the following equation:

$RI\%=\frac{\theta_{rev}- \theta_{d}}{\theta_{n}- \theta_{d}}$ (Eq. S6)

Where θ_n_ and θ_d_ are the protein CD signal at 222 nm in the native and denatured state (recorded respectively at 25°C and 90°C), θ_rev_ is the CD signal at 222 nm recorded at 25°C after the first heating scan.

1. **CD SPECTRA**


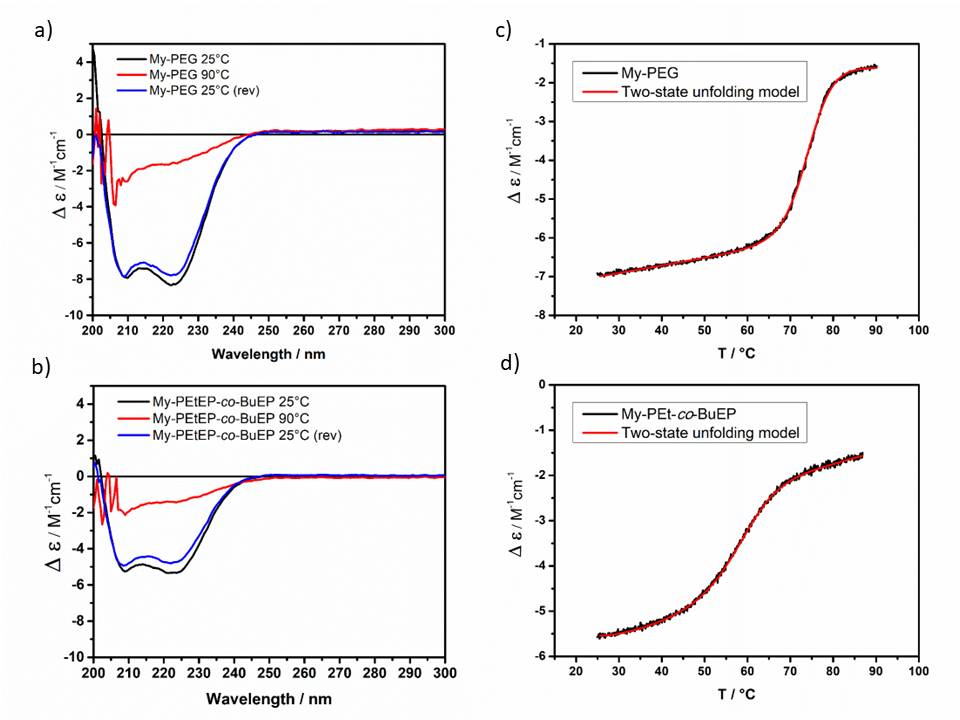


*Figure S1: CD spectra of a) My-PEG and b) My-PEtEP-co-BuEP at 25°C (black curve), 90°C (red curve), and at 25°C after cooling (blue curve). Thermal denaturation profilesvof (c) My-PEG and d) My-PEtEP-co-BuEP performed by VT-CD measurements, monitoring the CD signal at 222 nm during a heating scan at 1°C/min. The red line is the theoretical curve calculated using a two-state unfolding model.*


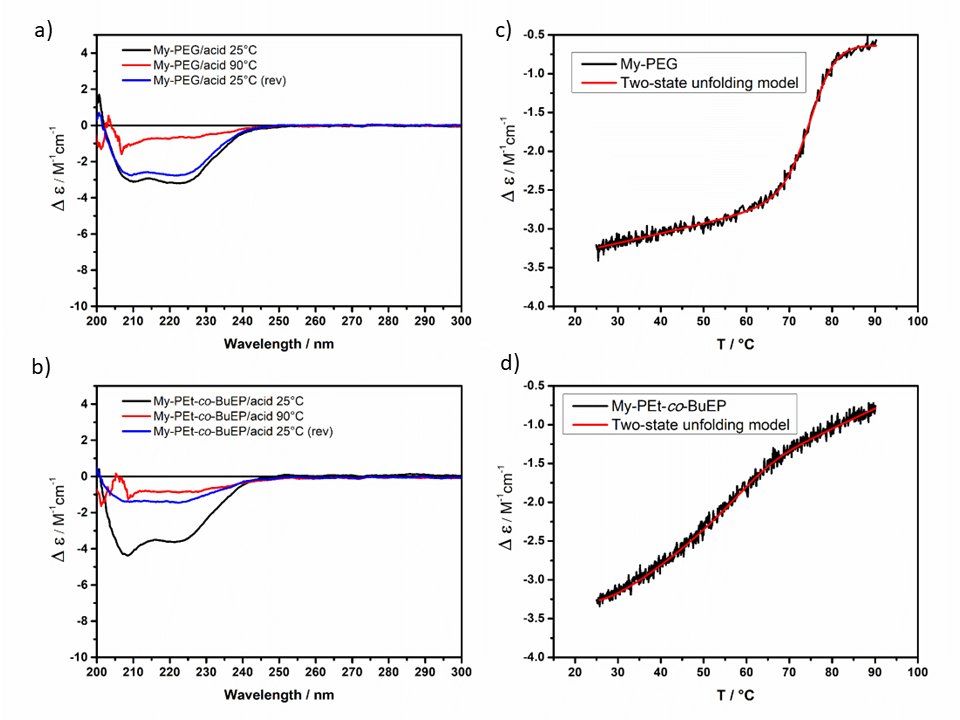


*Figure S2: CD spectra of a) My-PEG and b) My-PEtEP-co-BuEP at 25°C (black curve), 90°C (red curve), and at 25°C after cooling (blue curve) in presence of sulphuric acid . Thermal denaturation profiles (black line) of (c) My-PEG and (d) My-PEtEP-co-BuEP in presence of sulphuric acid (final sulfuric acid concentration: 1*10^-3^ M; molar ratio denaturant/protein 2*10^3^), performed by VT-CD measurements, monitoring the CD signal at 222 nm during a heating scan at 1°C/min. The red line is the theoretical curve calculated using a two-state unfolding model. In the case of My-PEtEP-co-BuEP in presence of sulfuric acid, being the RI % of only 18%, the data could not be treated with thermodynamic models, hence the fitting was performed only as a comparison with the other samples.*


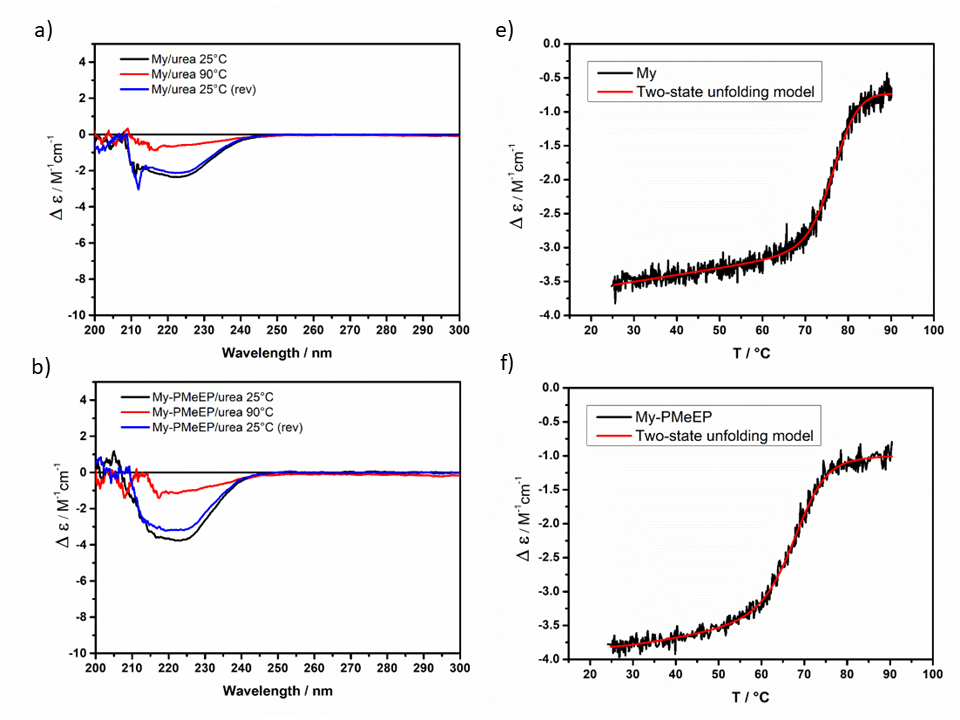

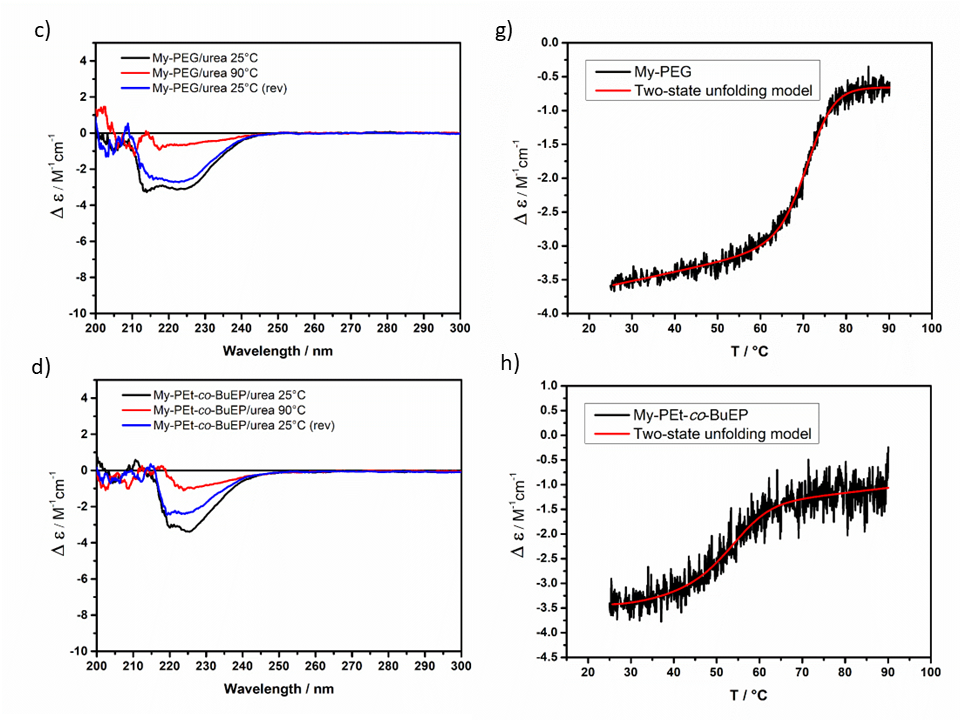


*Figure S3: CD spectra of a) My, b) My-PMeEP, c) My-PEG and d) My-PEtEP-co-BuEP at 25°C (black curve), 90°C (red curve), and at 25°C after cooling (blue curve), in presence of* *urea. Thermal denaturation profiles of e) My, f) My-PMeEP, g) My-PEG and h) My-PEtEP-co-BuEP in presence of urea (final urea concentration: 0.5 M; molar ratio denaturant/protein 5*10^5^), performed by VT-CD measurements, monitoring the CD signal at 222 nm during a heating scan at 1°C/min. The red line is the theoretical curve calculated using a two-state unfolding model.*

1. **REFERENCES**

(1) Greenfield, N. J. Using Circular Dichroism Spectra to Estimate Protein Secondary Structure. *Nat. Protoc.* **2007**, *1* (6), 2876–2890. https://doi.org/10.1038/nprot.2006.202.

(2) Greenfield, N. J. Determination of the Folding of Proteins as a Function of Denaturants, Osmolytes or Ligands Using Circular Dichroism. *Nat. Protoc.* **2006**, *1* (6), 2733–2741. https://doi.org/10.1038/nprot.2006.229.

(3) Greenfield, N. J. Using Circular Dichroism Collected as a Funcion of Temperature to Determine the Thermodynamics of Protein Unfolding and Binding Interactions. *Nat. Protoc.* **2006**, *1* (6), 2527–2535. https://doi.org/10.1038/nprot.2006.204.Using.

(4) Mehl, A. F.; Crawford, M. A.; Zhang, L. Determination of Myoglobin Stability by Circular Dichroism Spectroscopy: Classic and Modern Data Analysis. *J. Chem. Educ.* **2009**, *86* (5), 600–602. https://doi.org/10.1021/ed086p600.
